# Post-radiation lichen planus: a case report and review of the literature

**DOI:** 10.1186/s13256-024-04389-3

**Published:** 2024-03-31

**Authors:** Adam N. Musick, Do Young Kim, Steven J. Baumrucker

**Affiliations:** 1 MSIV, ETSU Quillen College of Medicine, Johnson City, TN USA; 2 Department of Oncology, ETSU-Quillen College of Medicine, Johnson City, TN USA; 3 Department of Palliative Medicine, Ballad Health System, Johnson City, TN USA

**Keywords:** Lichen planus, Radiation therapy, Cancer, Adverse event, Case report

## Abstract

**Background:**

Lichen planus is a T-cell mediated inflammatory disorder of the skin and mucus membranes and is a rare complication of external beam radiation.

**Case presentation:**

64 year old White male who presented to dermatology with a lesion at the lateral aspect of the right thigh. The lesion was first noted 40 years prior and had grown from 1.5 cm to 6.5 cm in the ensuing years. On examination the lesion was raised, hypopigmented, with pearly borders and central ulceration. Wide excision with lymph node dissection demonstrated invasive squamous cell carcinoma, basaloid type, with negative margins. Patient had radiation therapy of the right inguinal nodes. Patient subsequently noted a “blister” on the right upper thigh, which progressed over time to flat, polygonal, intensely pruritic lesions that covered the right lateral thigh from just distal to the hip to the distal third of the femur (Figs. 1, 2). Skin biopsy was positive for lichen planus. He was started on topical triamcinolone with salutary effects on appearance and pruritus.

**Fig. 1:**
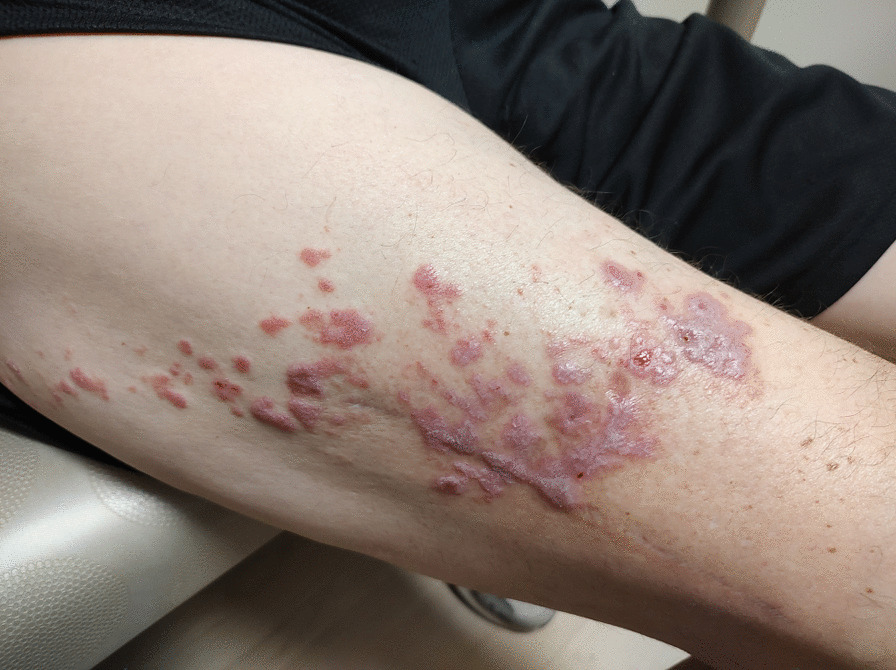
Lichen planus, right thigh

**Fig. 2:**
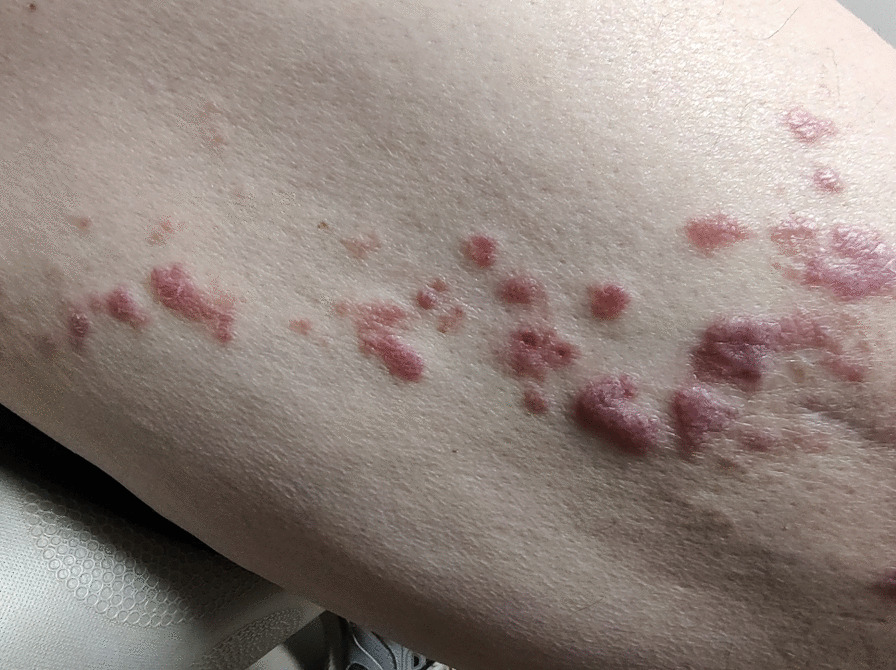
Closeup, lichen planus, right thigh, demonstrating polygonal papules

**Conclusion:**

Once more biopsy-proven cases of post-radiation lichen planus are reported, hopefully the exact mechanism can be elucidated. This may identify risk factors and aid in treatment, with the goal of limiting radiation toxicity and palliation of symptoms that may affect the quality of daily life.

## Background

This is a patient who presented with lichen planus, a rare adverse effect from external beam radiation. Between 2002 and 2017, only 12 cases were reported in the medical literature [1]. Because of its low incidence, providers should be informed to prevent delay in treatment or misdiagnosis.

## Case report

The patient is a 64 year old White male who presented to dermatology with a lesion at the lateral aspect of the right thigh. The lesion was first noted 40 years prior and had grown from 1.5 cm to 6.5 cm in the ensuing years. On examination the lesion was raised, hypopigmented, with pearly borders and central ulceration. Wide excision with lymph node dissection demonstrated invasive squamous cell carcinoma, basaloid type, with negative margins. Tumor cells were positive for CK5/6, CKAE1/AE3 and p40 immunostatins. Pathology from the right inguinal node was positive for metastatic squamous cell carcinoma. The patient has been treated with cetuximab, pembrolizumab, carbo/taxol, with progression, but stabilized on cimiplimab-rwlc. He had sterotactic ablative radiotherapy (SABR) to a single right lung nodule.

The patient had radiation therapy of the right inguinal nodes. He had chemoradiation which delivered 6000cGy to the right thigh and 5000cGy to the right inguinal and iliac nodes completed 1 year ago. Recently a CT scan of the chest, abdomen and pelvis showed treated lung metastases, persistent ground-glass nodule in the upper lobe and a recurrent 1.2 cm distal right para external iliac metastasis. He was symptomatic with right abdominopelvic pain. He had 3000cGy radiation to the distal right para-external iliac metastasis.

Patient subsequently noted a new “blister” on the right upper thigh, distinct from his previous squamous cell cancer, which progressed over time to flat, polygonal, intensely pruritic lesions that covered the right lateral thigh from just distal to the hip to the distal third of the femur (Figs. 1, 2). There were no oral or conjunctival lesions. He denied oral sensitivity. There were no other skin lesions noted, and nail beds were unremarkable. The rest of the physical exam was nondiagnostic. Skin biopsy demonstrated saw-tooth epidermal hyperplasia with wedge-shaped hypergranulosis and basilar vacuoles diagnostic for lichen planus. He was started on topical triamcinolone with salutary effects on appearance and pruritus within 1 week, which continued to improve over the next several months.

## Discussion

Lichen planus is a T-cell mediated inflammatory disease of the skin and mucus membranes [2]. It is typically described as “planar, purple polygonal pruritic papules/plaques” on the skin that are visually distinctive and often accompanied by whitish lines called Wickham striae [3].

### Presentation/etiology/triggers

Lichen planus is a mucocutaneous inflammatory disease of unknown origin [4] that most commonly affects the skin and oral mucosa [5]. Additional mucous membranes including the oral, vulvovaginal, esophageal, laryngeal, and conjunctival mucosa can also be affected and with different variants based on lesion morphology and site of involvement [6]. It can affect multiple areas either simultaneously or sequentially [7]. Cutaneous lichen planus is often pruritic and is characterized by flat-topped violaceous papules and may result in residual hyperpigmentation, specifically in dark-skinned individuals [8].

Oral lichen planus is a chronic disease that presents as symmetric white, lacelike network reticular lesions in addition to papules, plaques, erythematous lesions, and erosions [9]. Genital lichen planus demonstrates a wide range of morphological presentations, and in its erosive form, can result in significant scaring and pain [10].

Based on population data from Sweden, the prevalence of cutaneous lichen planus is 0.3% in males and 0.1% in females [11], whereas the prevalence of oral lichen planus is 1.5% in males and 2.3% in females [12]. Oral lichen planus has been considered premalignant and associated 1% incidence of squamous-cell carcinoma has been reported [13]. There have also been case reports in the literature describing cases of squamous cell carcinoma arising from anogenital, esophageal, and hypertrophic cutaneous lichen planus lesions [12, 14, 15].

### Pathophysiology

Lichen planus is one of several T-cell mediated autoimmune disorders of the skin (psoriasis and vitiligo being notable examples.) T-cells accumulate in the basal membrane, a phenomenon which is triggered by an aberrant and overabundant immunologic response to the death of keratin-containing cells, often from viral infections, trauma, chemical damage, or exposure to ultraviolet radiation. Keratocyte damage results in the release of Damage-Associated Molecular Patterns (DAMPs); these are recognized by dendritic cells that can trigger a cascade of production of inflammatory cytokines (e.g., interleukins, tumor necrosis factors, etc.). Such cytokine-rich environment promotes migration of T-cells to the area, inducing T-Cell mediated inflammation [16]. Genetic factors have been suggested to play a role in the disease through rare cases of familial lichen planus and the overexpression of specific HLA haplotypes including HLA-DR1 in cutaneous lichen planus [4].

### Treatment

Treatment of lichen planus depends on the location and severity of the lesions [3]. First-line treatment for all forms of lichen planus consists of high-potency topical corticosteroids and hypertrophic lesions are best treated with intralesional triamcinolone acetonide (Kenalog) [17]. Second-line therapy for treating genital and oral lichen planus includes topical calcineurin inhibitors, tacrolimus and pimecrolimus [18]. Severe widespread lichen planus is treated with prolonged oral prednisone therapy [19].

### Radiation-induced lichen planus

Although there have been reports in the literature of oral lichen planus developing after radiation therapy, cutaneous lichen planus arising post–radiation therapy is a rare finding in the English language literature. Currently there is a poor understanding of the specific role of ionizing radiation in the creation of lichen planus [20], but ultraviolet radiation exposure is known to be a risk factor [21].

In 1985, Yates *et al.* [22] proposed that the appearance of lesions in prior radiation fields could be an isomorphic, or Koebner, response from radiation injury. The isomorphic response of Koebner has been shown to occur often in lichen planus, and it is described as the appearance of lesions in regions of skin subjected to trauma [23]. Additionally, the isomorphic response can develop due to other forms of irritation including burns, lacerations, friction, and ultraviolet light [24].

Shurman *et al.*, suggested the term “isoradiotopic response” to describe the occurrence of secondary dermatoses appearing in radiation fields. Kluger *et al.*, [1] proposed that radiation-induced lichen planus is most likely due to patient’s receiving X-rays or gamma ray irradiation and is less likely due to electron therapy, and showed that the median onset of radiation-induced lichen planus was estimated to be 30.7 days. Despite the above, the role of ionizing radiation in the development of lichen planus remains poorly understood.

## Conclusion

We present a case of post-radiation lichen planus, a rare dermatologic complication that is still poorly understood in its pathophysiology in relation with the role of ionizing radiation therapy. Clinical management often entails symptomatic management including the use of topical steroid and calcineurin inhibitors, and oral steroids in severe cases. In this case, the patient achieved remission of the lichen planus with topical triamcinolone application. Recurrence is prevalent but often with less severity [24], and the overall treatment plan does not differ. Once more biopsy-proven cases of post-radiation lichen planus are reported, hopefully the exact mechanism can be elucidated. This may identify risk factors and aid in treatment, with the goal of limiting radiation toxicity and palliation of symptoms that may affect the quality of daily life.

## Data Availability

N/a.
